# Commentary: Monetary, Food, and Social Rewards Induce Similar Pavlovian-to-Instrumental Transfer Effects

**DOI:** 10.3389/fnbeh.2017.00136

**Published:** 2017-07-20

**Authors:** Sara Garofalo, Giuseppe di Pellegrino

**Affiliations:** ^1^Department of Psychiatry, University of Cambridge Cambridge, United Kingdom; ^2^Department of Psychology, Center for Studies and Research in Cognitive Neuroscience, University of Bologna Bologna, Italy

**Keywords:** the Pavlovian-to-instrumental transfer, awareness, reward, subliminal priming, unconscious processing

In their report, Lehner et al. (2017) adopt the Pavlovian-to-Instrumental Transfer (PIT) paradigm to compare the ability of different rewards to drive behavior. The PIT-tests the extent to which a Pavlovian cue (i.e., a reward-associated cue) can elicit instrumental responses independently paired with the same (specific PIT), or a similar (general PIT), reward (Garofalo and di Pellegrino, 2016). During PIT-test, instrumental responses—previously associated with a reward—are performed under extinction, while task-irrelevant Pavlovian cues are concurrently presented. The crucial question is: can an external stimulus that recalls a reward, modulate reward-seeking behavior? Such a mechanism has both adaptive (e.g., effectively providing for needed food), and maladaptive implications (e.g., looking for food even when not necessary; see Everitt et al., 2001).

Even if awareness of such Pavlovian influences is not the main focus of their study, the authors indirectly introduced the idea that PIT can be the result of an explicit strategy. They claim that reward-predicting stimuli (i.e., Pavlovian cues) can consciously or unconsciously exert a strong influence on behavior, and go further directly asking participants whether or not they pursued a conscious strategy during PIT. A conscious strategy here refers to participants explicitly referring that their choice—during the PIT phase—was related to the identity of the Pavlovian stimulus. The reported results suggest that participants who declared to have used a conscious strategy during PIT also exhibited a higher general PIT effect. But is there room for awareness in PIT?

The question whether there is a conscious or unconscious influence of Pavlovian cues on behavior is crucial for understanding the PIT effect, and some clarifications need to be pointed out regarding the different interpretations that the word “awareness” has in learning literature. Awareness can, indeed, be differentially intended as awareness of the *stimuli*, awareness of the *contingency*, or awareness of the *strategy*.

*Awareness of the stimuli* refers to the possibility that subliminal (i.e., not consciously perceived) stimuli can influence behavior. Studies showed how instrumental responses can be conditioned even if the outcomes are subliminal (Pessiglione et al., 2008). Furthermore, there is evidence for sensory-specific satiety when external stimuli are not consciously perceived (Ziauddeen et al., 2012). However, subliminal Pavlovian conditioning is more controversial (see Lovibond and Shanks, 2002 for a review).

*Awareness of the contingency* refers to the explicit learning of the association between a stimulus (e.g., a picture) or a response (e.g., a button press) with its associated reward (e.g., the blue fractal is followed by a 1€ win). Contingency learning is an essential precondition for PIT and must be achieved in order to make any claim about how learned associations may subsequently interact. But if such acquisition is either aware (e.g., the participants may explicitly refer to the learned associations) or unaware (e.g., the Pavlovian stimulus implicitly induces an arousal) should make no difference for the interpretation of the PIT effect.

In a series of studies, Hogarth et al. (2007, 2010), Trick et al. (2011) investigated the relationship between PIT and contingency awareness, and found that only participants able to explicitly report knowledge of the stimulus–reward contingency were influenced by the Pavlovian stimulus itself. This result was interpreted as an evidence for a cognitive account of the PIT effect, over a habit account. Pavlovian cues enhanced reward seeking by influencing explicit decision-making, rather than implicit associations (Hogarth et al., 2010). However, in these studies, participants who are unaware of the contingencies are usually excluded from the analysis, and there is no clarification about a possible implicit manifestation of learning. In one of our studies on patients with ventromedial prefrontal cortex lesions, we found the presence of a PIT effect even if no evidence of explicit Pavlovian conditioning was observed (unpublished data). To correctly conclude that awareness effectively plays a key role in reward-seeking, it is crucial to assess that contingency learning has taken place. Only then a contrast between aware and unaware learners is possible. If there is no learning at all, it is simply impossible to expect any interaction or transfer between two forms of learning that did not occur in the first place. Why should an otherwise neutral stimulus trigger any kind of goal-directed behavior, if not because it is predictive of a reward?

*Awareness of the strategy* is intended as the awareness that the exposure to a specific Pavlovian cue is determining the instrumental choice performed. This kind of awareness can give birth to two possible interpretations of the PIT effect: (1) a pure and simple Pygmalion effect, in which participants understand the experimenter's expectation and conform their performance to such expectation; (2) a cognitive priming coming from a consciously perceived external stimulus.

While the first interpretation may be wisely pursued—according to Occam's razor principle to favor the simplest interpretation of a phenomenon—the second one opens to different possibilities.

Following the standard economic theory, a rational behavior should neglect the Pavlovian stimulus, as it is not informative about the instrumental task (Huys et al., 2011), and no reinforcers are available (PIT is under extinction). Huys and colleagues argued that, if a conscious strategy is used in PIT, then Pavlovian influences extend even into cognitive choices (Huys et al., 2011). Lehner et al. (2017) results seem to be interpreted in this direction.

But is it really possible to disentangle implicit and explicit influences of Pavlovian cues? Evidence of various forms of self-reported awareness still does not rule out the possibility that PIT is also—if not only—supported by an implicit process. Even if it is possible to dissociate them, the possibility of a simple Pygmalion effect should still be considered.

A unifying view may consist in the hypothesis that PIT, at least in humans, has two components: an implicit component, possibly ascribed to subcortical structures like amygdala and striatum (Corbit and Balleine, 2005; Corbit et al., 2007; Talmi et al., 2008); and an explicit component, more cognitive and strategic, possibly ascribed to more cortical frontal areas (Holmes et al., 2010). In this scenario, can the first component be more prone to evolve in a maladaptive behavior (e.g., drug-seeking), as compared to the second one?

## Author contributions

SG conceived the main idea presented and wrote the manuscript. GdP significantly contributed to the scientific discussion on the topic and revised the manuscript.

### Conflict of interest statement

The authors declare that the research was conducted in the absence of any commercial or financial relationships that could be construed as a potential conflict of interest.

## References

[B1] Corbit L. H. Balleine B. W. (2005). Double dissociation of basolateral and central amygdala lesions on the general and outcome-specific forms of pavlovian-instrumental transfer. J. Neurosci. 25, 962–970. 10.1523/JNEUROSCI.4507-04.2005 15673677 PMC6725628

[B2] Corbit L. H. Janak P. H. Balleine B. W. (2007). General and outcome-specific forms of Pavlovian-instrumental transfer: the effect of shifts in motivational state and inactivation of the ventral tegmental area. Eur. J. Neurosci. 26, 3141–3149. 10.1111/j.1460-9568.2007.05934.x 18005062

[B3] Everitt B. J. Dickinson A. Robbins T. W. (2001). The neuropsychological basis of addictive behaviour. Brain Res. Rev. 36, 129–138. 10.1016/S0165-0173(01)00088-1 11690609

[B4] Garofalo S. di Pellegrino G. (2016). Individual differences in the influence of task-irrelevant Pavlovian cues on human behavior, in Neural Circuitry of Behavioral Flexibility: Dopamine and Related Systems, eds Bissonette G. B. Roesch M. R. (Frontiers Media SA), 74–84. Available online at: http://www.frontiersin.org/books/Neural_Circuitry_of_Behavioral_Flexibility_Dopamine_and_Related_Systems/833 10.3389/fnbeh.2015.00163 PMC4478391 26157371

[B5] Hogarth L. Dickinson A. Duka T. (2010). The associative basis of cue-elicited drug taking in humans. Psychopharmacology 208, 337–351. 10.1007/s00213-009-1735-9 19960187

[B6] Hogarth L. Dickinson A. Wright A. Kouvaraki M. Duka T. (2007). The role of drug expectancy in the control of human drug seeking. J. Exp. Psychol. Anim. Behav. Process. 33, 484–496. 10.1037/0097-7403.33.4.484 17924795

[B7] Holmes N. M. Marchand A. R. Coutureau E. (2010). Pavlovian to instrumental transfer: a neurobehavioural perspective. Neurosci. Biobehav. Rev. 34, 1277–1295. 10.1016/j.neubiorev.2010.03.007 20385164

[B8] Huys Q. J. M. Cools R. Gölzer M. Friedel E. Heinz A. Dolan R. J.. (2011). Disentangling the roles of approach, activation and valence in instrumental and Pavlovian responding. PLoS Comput. Biol. 7:e1002028. 10.1371/journal.pcbi.1002028 21556131 PMC3080848

[B9] Lehner R. Balsters J. H. Herger A. Hare T. A. Wenderoth N. (2017). Monetary, food, and social rewards induce similar pavlovian-to-instrumental transfer effects. Front. Behav. Neurosci. 10, 1–12. 10.3389/fnbeh.2016.00247 28101010 PMC5209382

[B10] Lovibond P. F. Shanks D. R. (2002). The role of awareness in Pavlovian conditioning: empirical evidence and theoretical implications. J. Exp. Psychol. Anim. Behav. Process. 28, 3–26. 10.1037//0097-7403.28.1.3 11868231

[B11] Pessiglione M. Petrovic P. Daunizeau J. Palminteri S. Dolan R. J. Frith C. D. (2008). Subliminal instrumental conditioning demonstrated in the human brain. Neuron 59, 561–567. 10.1016/j.neuron.2008.07.005 18760693 PMC2572733

[B12] Talmi D. Seymour B. Dayan P. Dolan R. J. (2008). Human pavlovian-instrumental transfer. J. Neurosci. 28, 360–368. 10.1523/JNEUROSCI.4028-07.2008 18184778 PMC2636904

[B13] Trick L. Hogarth L. Duka T. (2011). Prediction and uncertainty in human Pavlovian to instrumental transfer. J. Exp. Psychol. Learn Mem. Cogn. 37, 757–765. 10.1037/a0022310 21319920

[B14] Ziauddeen H. Subramaniam N. Gaillard R. Burke L. K. Farooqi I. S. Fletcher P. C. (2012). Food images engage subliminal motivation to seek food. Int. J. Obesity 36, 1245–1247. 10.1038/ijo.2011.239 22143617 PMC3438467

